# Effects of dynamic aging on the wear and fracture strength of monolithic zirconia restorations

**DOI:** 10.1186/s12903-018-0618-z

**Published:** 2018-08-23

**Authors:** Işıl Sarıkaya, Yeliz Hayran

**Affiliations:** Department of Prosthodontics, Tokat Gaziosmanpasa University Faculty of Dentistry, 60100 Tokat, Turkey

**Keywords:** Dynamic aging, Fracture strength, Monolithic zirconia, Wear

## Abstract

**Background:**

The purpose of this study is to evaluate the wear and fracture strength of crowns and three-unit partial fixed dental prosthesis (FDP) fabricated using by Bruxzir and Incoris TZI as recently introduced monolithic zirconia materials.

**Methods:**

A total of sixteen crowns and sixteen three-unit FDPs were fabricated using Bruxzir and Incoris TZI (*n* = 8). All specimens were subjected to a 2-body wear test in a dual axis chewing simulator for 1,200,000 loading cycles against steatite antagonist balls. The fracture strength and volumetric loss were recorded. The obtained data were statistically analyzed by 2-way ANOVA testing (α = 0.05).

**Results:**

The mean volumetric loss of the crowns was higher than that of the three-unit FDPs (*p* < 0.05). Of the two monolithic systems, Incoris TZI exhibited more wear than Bruxzir. The fracture strengths of Bruxzir crowns and FDPs were found to be higher than those of the crowns and FDPs fabricated with Incoris TZI (*p* < 0.05).

**Conclusion:**

In in vitro test conditions, Bruxzir and Incoris TZI monolithic zirconia systems are fracture-resistant for the crown and FDP application against physiologic chewing forces owing to dynamic aging. Among newly developed monolithic zirconia materials, Bruxzir is found to be more resistant to fracture compared to the Incoris TZI.

## Background

Esthetic expectations are the main reason for preferring ceramic restorations, for which the usual processing method is veneering. Major problems associated with multilayered restorations are their low fracture strength and surface chipping. Therefore, new processing techniques have been developed to resolve the chipping problem encountered with ceramic veneering layers [1]. For example, to eliminate the porosity generated within the veneering layer, injection of porcelain over the zirconia framework can be carried out [1]. In addition, CAD-on and rapid layer techniques have become popular in recent years in prosthetic dentistry. Developments in CAD-CAM (computer-aided design, and computer-aided manufacturing) technology have also increased the diversity of materials that can be used for restorations. In this context, new materials, such as PICN (polymer infiltrated ceramic network) materials and monolithic ceramics, are available today for use.

Monolithic restorations aim at improving the final quality of restorations. Further, the problems of surface flaws and chipping problems encountered with veneering can be resolved using monolithic zirconia restorations [2]. Zirconia restorations exhibit good mechanical properties, such as high flexural strength along with good esthetic characteristics and biocompatibility. In order to achieve good results with restorations, the wear properties of restorations should be similar to those of human enamel [3]. Furthermore, restorations should be conservative for antagonist dentition. Although short-term data is available on high-strength zirconia systems, research is still needed on periodontally weakened teeth and bruxism [2].

Physiologic chewing forces are in the range of 10–120 N, while parafunctional forces are greater in the range of 200–800 N [4–8] which both affect biomaterial survival. Apart from the chewing characteristics and force configuration, clinical parameters, such as moisture, temperature, and pH, also influence the mechanical properties and behavior of materials in the oral cavity [9]. Since the 1940s, chewing-mimicking devices are being used for determining the occlusal wear of restorative materials [10]. Various in-vitro wear tests have been developed to simulate clinical conditions since then. The dual-axis chewing simulator developed by Willytech is often considered as a precise instrument for the fatigue testing of dental materials [10]; several research groups have investigated the wear performance and fracture strength of ceramics with chewing simulators [11–20]. Even today, research is ongoing for new simulators for the preclinical testing of dental materials in vitro chewing simulation conditions [10, 16, 21].

Monolithic zirconia restorations are not preferred when the esthetic function is the priority. However, these systems are beneficial in the case of fixed dental prostheses supported by pathological attrition or severely damaged teeth (FDPs). Adhesive bonding of monolithic restorations is beneficial in various clinical situations, such as excessive unloading forces, compromised mechanical retention, and limited space for adequate tooth preparation [22]. Furthermore, the resin bonding of zirconia restorations is advocated for improving the fracture strength of restorations [22, 23].

The high fracture strength of yttria-stabilized zirconia (YSZ) is attributed to the physical properties of partially stabilized zirconia. In previous studies, the fracture strength of YSZ was reported to vary from 900 N [24] to 2000 N under static loading [25, 26].

Preclinical evaluations help to determine the physical and mechanical behavior of materials. Although the fatigue testing standards (DIN EN ISO 22674) of fixed dental prosthesis materials are established under certain test conditions, it is controversial how much of the intraoral conditions are accurately represented by these standards [27, 28]. Restoration fatigue behavior is required to provide reliable data on the strength characteristics of materials. Usually, universal testing machine data on the fatigue behavior of tested materials are used but oral thermal conditions are not included in this testing.

The aim of this in vitro study is to evaluate the wear and fracture strength of crowns and three-unit partial FDPs fabricated using recently introduced monolithic zirconia materials and subjecting them to 1,200,000 chewing cycle versus steatite balls. The null hypothesis tested was that no difference would be detected in the wear and fracture strength properties of different tested materials.

## Methods

### Preparation of specimens

In the present study, a mandibular left first molar tooth of the dentulous mandibular cast (Frasaco AG-3 GmbH, Tettnang, Germany) was selected for producing monolithic crown restorations. A mandibular left second premolar tooth (Frasaco AG-3 GmbH, Tettnang, Germany) and a mandibular left second molar tooth (Frasaco AG-3 GmbH, Tettnang, Germany) were selected for fabricating the FDPs. The selected teeth were prepared according to the accepted tooth preparation principles using a chamfer diamond rotary instrument (229-014XC Torpedo, Romidan, Kiryat-Ono, Israel) by adjusting for a 1 mm circumferential chamfer margin, 1.5 mm occlusal reduction, 1 mm axial preparation, and 6° convergence angle. After preparation, the master casts were evaluated using a surveyor to detect undercuts. The prepared teeth were then duplicated as master dies made of Ni-Cr by laser sintering. In total, thirty-two master model dies were obtained, including sixteen master casts that were made as crowns and sixteen master casts that were made as three-unit FDPs; the model dies were fabricated with Bruxzir (Glidewell Laboratories, CA, USA) and Incoris TZI (Sirona Dental Systems GmbH, Bensheim, Germany) (*n* = 8). Bruxzir crowns and three-unit FDPs were fabricated using monolithic zirconium blanks (Bruxzir Solid Zirconia Milling Blanks, 98,5 × 20 mm, Glidewell Laboratories, CA, USA) designed using a Cerec inLab MC X5 system (Sirona Dental Systems GmbH, Bensheim, Germany). Incoris TZI crowns and three-unit FDPs were fabricated from monolithic blocks (40/19 = 40x19x15.5 mm) and designed using a Cerec inLab MC X5 system (Sirona Dental Systems GmbH, Bensheim, Germany). The chemical composition, according to the manufacturer’s declaration of investigated Y-TZP ceramics is shown in Table 1. A connector size of 9 mm^2^ was selected for FDPs as recommended by the manufacturers. Bruxzir restorations were sintered at a temperature of 1580 °C for 2 h and then glazed with Bruxzir spray glaze powder (Glidewell Laboratories, CA, USA) at a temperature of 830 °C according to the manufacturer’s instructions. Incoris TZI restorations were sintered at a temperature of 1510 °C for 2 h and then glazed with Cerec speed glaze spray (Sirona Dental Systems GmbH, Bensheim, Germany) at a temperature of 750 °C according to the manufacturer’s instructions. All the restorations and preparations were carried out by the same dentist. Eight crowns and FDPs were created with the two different zirconia materials randomly.

**Table 1:** Chemical composition of the Y-TZP dental ceramics expressed as weight percent (wt.%)

Ceramic	wt.%						
	Y_2_O_3_	HfO_2_	Al_2_O_3_	SiO_2_	Fe_2_O_3_	Na_2_O	ZrO_2_
Bruxzir	4.1	4.0	0.34	< 0.01	< 0.01	< 0.01	Balance
Incoris TZI	4.5–6.0	< 5.0	< 0.05	< 0.05	< 0.05	< 0.05	Balance

### Luting of the crowns

All the restorations were adhesively luted on Ni-Cr master cast dies using a dual cure composite material (Panavia F 2.0, Kuraray Medical Co., Tokyo, Japan) according to the manufacturer’s instructions. The master cast dies were sun-blasted with 50 μm Al_2_O_3_ powder at an air pressure of 2.5 bar for 10 s. Equal amounts of Panavia Paste A and B (Panavia F 2.0, Kuraray Medical Co., Tokyo, Japan) were mixed and applied to the intaglio surfaces of the restorations according to the manufacturer’s instructions. The restorations were seated onto the dies and held in place by the application of finger pressure. Subsequently, the restorations were cured using a curing light for 20 s. Excess cement was removed with sponge pellets before curing and an air-blocking gel (Oxiguard II, Kuraray Medical Co., Tokyo, Japan) was applied during the setting of the resin cement over 3 min. The obtained specimens were stored for 24 h at 37 °C before being subjected to dynamic aging.

### Dynamic aging

**Fig. 1:**
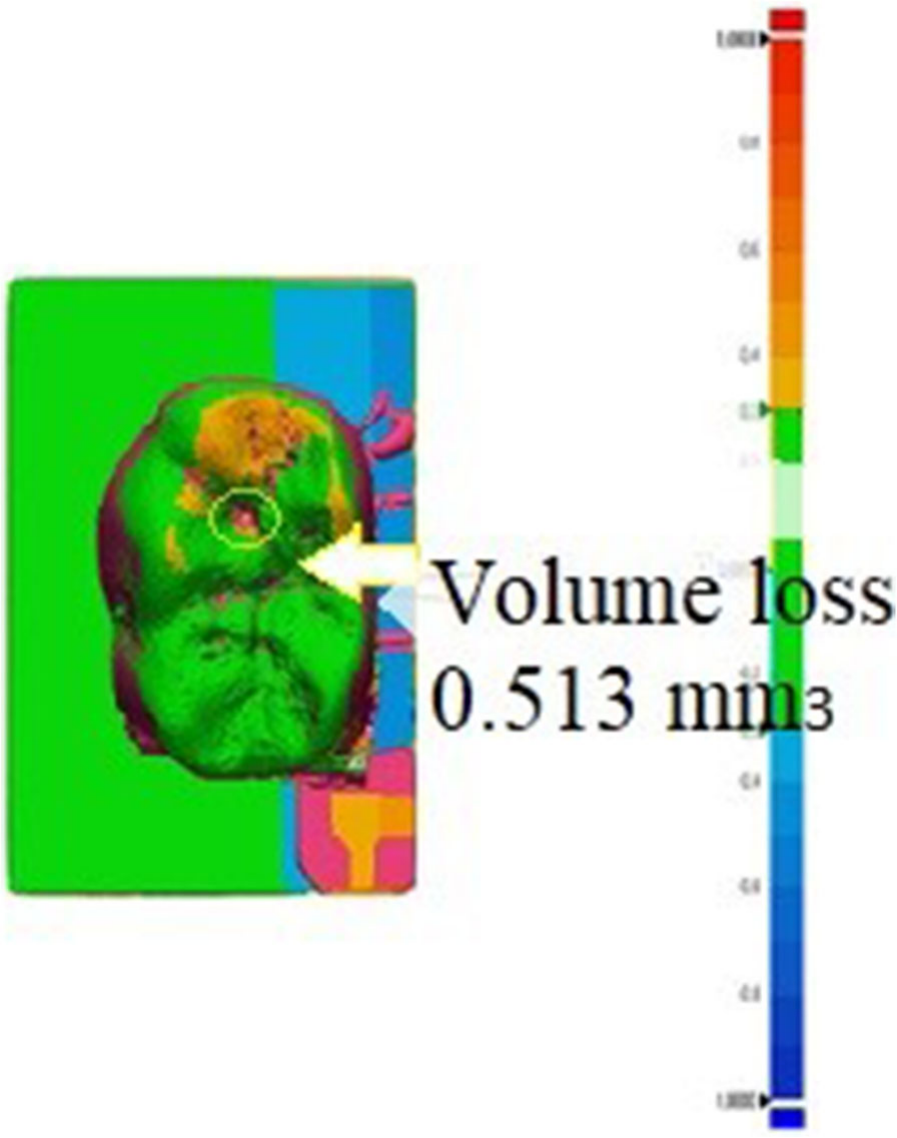
Laser scanner image of the specimen’s with 3-dimensional surface analysis, and the volumetric loss (mm^3^)

All the root surfaces of the metal dies were coated with a 1 mm-thick polyether layer (Impregum Soft, 3 M Espe, St Paul, MN, USA) from the marginal finish line of the restorations to 2-mm apical direction for the purpose of simulating the physiologic mobility of teeth. The metal dies were immersed in a wax bath, which was replaced by polyether in a second fabrication process, as previously described (17,18). Later, restorations on master cast dies were fixed in a resin mold, which acts as the sample holder for the chewing simulator, using a self-curing acrylic resin material (Meliodent, Heraeus Kulzer, Wehrheim, Germany). The specimens underwent thermocycling for 10,000 cycles between 5 and 55 °C over a dwell time of 60 s and a transfer time of 10 s (SD Mechatronik Thermocycler, SD Mechatronik GmbH, Feldkirchen-Westerham, Germany). After thermocycling, the specimens were subjected to a 2-body wear test in a dual axis chewing simulator (CS 4.2, SD Mechatronic GmbH, Feldkirchen-Westerham, Germany). Steatite ceramic balls (Höchst Ceram Tec., Wunsiedel, Germany) of 6 mm diameter were used as the antagonistic abraders. The balls were fixed to the upper sample holders of the chewing simulator using a light-curing composite resin (GC Pattern Resin, GC Corp., Tokyo, Japan). The chewing simulation parameters used are summarized in Table 2. The load was transferred to the center of the central fossa of the mandibular first crowns by opposing steatite balls. To simulate 5 years of clinical service, a total of 1,200,000 cycles were performed (9,10,12). After a 3-dimensional surface analysis using a laser scanner (LAS 20, SD Mechatronic GmbH, Feldkirchen-Westerham, Germany), the volumetric loss (mm^3^) in all the specimens was calculated (Fig. 1).

**Table 2:** The configuration of parameters set for dynamic aging

Parameter	Data
Number of cycles	1.200.000
Force	49 N
Height	3 mm
Lateral movement	1 mm
Descendent speed	60 mm/s
Lifting speed	60 mm/s
Feed speed	40 mm/s
Return speed	40 mm/s
Frequency	1.6 Hz

### Fracture strength test

Following the aging procedure, the specimens were tested on a universal testing machine (AGS-X, Shimadzu, Kyoto, Japan) until fracture. They were subjected to a compressive force at a crosshead speed of 1 mm/min with a round shaped modified bur of 4 mm diameter. A metal bar was positioned parallel to the long axes of the crown specimens and the buccal and lingual cusps of the crowns were used to apply the force. Force was transferred to the occlusal connector area of the FDP specimens. The maximum load necessary to fracture each specimen was recorded in Newtons (N).

### SEM

To characterize the surface wear patterns, selected specimens were evaluated by a scanning electron microscopy (SEM, Zeiss LEO 440, Oberkochen, Germany), for which the sample surfaces were initially coated with a thin layer of gold. The surfaces were then examined at a magnification of 100X at 25 keV.

### Statistical analysis

Statistical analysis was performed using SPSS 20.0 (IBM SPSS Statistics 20, IBM Co., Chicago, IL, USA) for Windows. Having assessed that all the obtained results were normally distributed, the wear and fracture load data were analyzed by two-way ANOVA. Bonferroni adjustment was used for multiple comparisons. Two methods and two monolithic zirconias were used for 4 groups with 80% power, 5% margin of error and effect size of 0.65 with 8 samples in each group, totaling 32 samples. The sampling volume was obtained with the help of the program G * power 3.1.2. The results are expressed as a mean ± standard deviation and the level of significance is set at 5% (*p* < 0.05).

## Results

### Wear

The mean volumetric loss (mm^3^) of the monolithic zirconia specimens is shown in Table 3. Two-way ANOVA showed no statistically significant differences when the wear values of Bruxzir and Incoris TZI crowns after 1,200,000 chewing cycles were analyzed (F = 10.874 and *p* = 0.003). The mean volumetric loss of the crowns was observed to be higher than that of three-unit FDPs (*p* < 0.05). Of the two tested monolithic systems, Incoris TZI exhibited more wear than Bruxzir.

**Table 3:** Mean values and standard deviations (SD) for volumetric loss (mm^3^) of the monolithic zirconias

	Crowns	FDPs	Total
Bruxzir	1,43 ± 0,12(a,x)	1,15 ± 0,17(a,y)	1,29 ± 0,21(a)
Incoris TZI	1,55 ± 0,11(a,x)	1,37 ± 0,16(b,y)	1,46 ± 0,16(b)
Total	1,49 ± 0,13(x)	1,26 ± 0,2(y)	1,38 ± 0,2

### Fracture strength

**Fig. 2:**
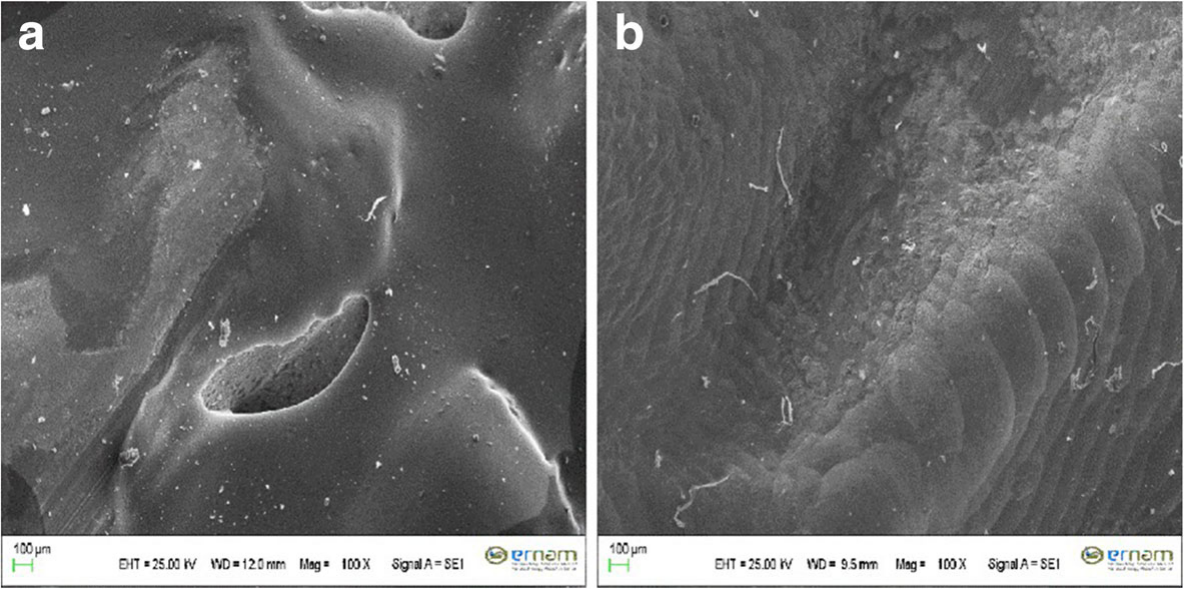
**a** Exemplary SEM picture (Magnification: 100×) of worn surface of a Bruxzir crown after dynamic aging. **b** Exemplary SEM picture (Magnification: 100×) of worn surface of an Incoris TZI crown after dynamic aging

None of the samples fractured during dynamic aging. The mean fracture strength (N) of the monolithic zirconia is shown in Table 4. According to the two-way ANOVA results, Bruxzir crowns exhibited significantly higher fracture strengths (4495 ± 221.33 N) than Incoris TZI crowns (3566.5 ± 217.24 N) (*p* < 0.05). Moreover, Bruxzir FDPs exhibited significantly higher fracture strengths (4506.25 ± 166.44 N) than Incoris TZI FDPs (3327.13 ± 185.81 N) (*p* < 0.05). Besides, no statistically significant differences could be observed between the Bruxzir crowns and FDPs (*p* > 0.05). Representative SEM images of the Bruxzir and Incoris TZI crowns are shown in Fig. 2a and b.

**Table 4:** Mean values and standard deviations (SD) for fracture load (N) of the monolithic zirconias

	Crowns	FDPs	Total
Bruxzir	4495,00 ± 221,33(a,x)	4507,25 ± 166,44(a,x)	4501,13 ± 189,29(a)
Incoris TZI	3566,5 ± 217,24(b,x)	3327,13 ± 185,81(b,y)	3446,81 ± 231,12(b)
Total	4030,75 ± 524,2(x)	3917,19 ± 632,79(y)	3973,97 ± 574,49

## Discussion

This in vitro study evaluated the wear and fracture strength of crowns and FDPs fabricated using two recently introduced monolithic zirconia materials. The null hypothesis tested in the present study, which assumed no difference in terms of the wear and fracture strength properties between the two tested materials, was rejected.

Zirconia has been developed with the aim of providing a stronger material for prosthetic dentistry. Ideal restorative materials should exhibit wear properties similar to those of human enamel and should not cause excess antagonist wear. Although short-term data is available on zirconia FDPs, a recent study showed that monolithic polished zirconia crowns caused less wear on antagonist enamel than glazed ceramic metal crowns [29]. In a study on the wear properties of dental ceramics, D’Archangelo et al. [15] reported that the volumetric loss values of IPS e.max Press (0.459 mm^3^), IPS e.max CAD (0.355 mm^3^), and Vita Mark II (0.472 mm^3^) were similar to that of human enamel (0.393 mm^3^). However, in the present study, 6 mm-thick disk-shaped specimens and a zirconia antagonist abrader were used. Moreover, the ceramic materials tested in this study exhibited lower hardness than Bruxzir and Incoris TZI monolithic systems.

Parafunctional chewing forces are approximately ten times greater than physiologic chewing forces [4–8]. Day bruxism is reported to affect 20% of the adult population and this number has increased over the past few decades [30]. In patients with bruxism, occlusal wear might be severe and fracture risk of the prosthesis might increase. Therefore, high strength restorative materials resistant to wear and fracture might be required, especially in the posterior region. However, the selected material should not cause temporomandibular joint disorders (TMJ) or increase the degree of dysfunction. Both the monolithic zirconia materials tested in the present study exhibited minimal volumetric loss at their ultimate strength.

The dynamic aging and fracture resistance of monolithic zirconia systems were determined by loading crowns and three-unit FDPs using an SD mechatronic chewing simulator (CS 4.2, SD Mechatronic GmbH). Heintze et al. [16] reported that the SD mechatronic chewing simulator is an adequate and cost-effective tool to test the fatigue strength of layered porcelain fused to metal crowns. The fracture strengths of 3-unit FDPs of different all-ceramic materials were tested using different forces up to 200 N [31–35]. Functional chewing forces were applied to the specimens (49 N) during the fracture strength test in the present study. The chewing force and characteristics can be changed individually [21]. Under bruxism conditions, teeth are subjected to larger forces over large lateral movement distances [13]. In terms of force configuration, dynamic aging analysis conducted in the present study was carried out in a manner similar to previous studies considering regular occlusal forces and bruxism [10, 15, 16].

It has been reported that almost all materials that have any geometrical shape such as composite, natural teeth, metal, ceramic or steatite can be examined with LAS 20 laser scanner [36]. Advanced users have the possibility to configure many sensor parameters. This includes, for example, median filtering in order to better highlight structures or the setting of the measurement gain in order to maintain the penetration depth of light into the material – and as such the scatter – as low as possible. This allows even the most difficult surfaces such as high gloss ceramics to be analyzed. After laser scanning, the Geomagic Software System allows us to import and export in different CAD data formats and analysis can be carried out beforehand/after comparison scans with matching, 3D-comparison, and 3D-PDFs. 3D analysis of the two scans along with abrasion depth can be seen on a color scale. Preis et al. [14] investigated the two-body wear performance of monolithic dental ceramics subjected to different surface treatments. They determined the vertical substance loss of different CAD/CAM ceramics and used a Laserscan 3D device as an optical profilometer. D’Archangelo et al. [15] used a CAD/CAM Contact Scanner for 3D surface analysis, wear depth, and volumetric loss of ceramics. Laser scanning in prosthetic dentistry is usually used to investigate marginal and internal fit of crown restorations [37]. In the present study, 3D laser images were supported by SEM images.

D’Archangelo et al. [15] reported that when human enamel cusps are used in vitro as antagonistic abraders, standardization of the study might be weak. In this context, steatite balls have been successfully used in the past [14, 17–19]. However, steatite balls cannot accurately mimic the complex enamel structure [14]. In order to overcome this disadvantage of the material, the steatite balls with the closest hardness property to enamel were used in the present study.

On the basis of the obtained findings, almost all the tested monolithic zirconia materials exhibited high load strengths. In a previous study, the fracture strength of YSZ was reported to be in the range of 900–1200 N [24]. In another study, the fracture strength of YSZ-FDPs was reported to be over 2000 N under static loading [25]. Eroğlu et al. [26] studied the fatigue behavior of zirconia-ceramic and reported a fracture strength of 2333 N for three-unit FPDs. Each specimen was subjected to 100,000 chewing cycles at a 50 N load and a 0.5 Hz frequency on the pontic with a 16 mm^2^ connector size. No specimen fractured during dynamic loading, similar to the present study. The dimensions of the connector area are crucial for determining the strength of FDPs. In the current study, a connector size of 9 mm^2^ was selected according to the manufacturer’s suggestion. Apart from the connector design [38], the fracture strength of three-unit FDPs is affected by several factors, such as the FDP location [12], tested chewing parameters, die materials [38], and used antagonist abraders [16]. In the present study, all the monolithic crowns and FDPs were adhesively luted on standardized laser sintering milled-Ni-Cr metal dies instead of polymethyl methacrylate (PMMA) dies. Further, dynamic aging defined in the present study was carried out in a manner similar to previous studies [10, 15, 16, 31, 34].

The major limitation of this study is the difficulty of determining the ideal chewing cycle. In this regard, Özcan and Jonasch [20], in a systematic review on the mechanical durability of all-ceramic single crowns and FDPs, reported that cyclic loading of restorations reduced the material-specific inclination and static fracture strength. However, there is no information on the fracture strength of the currently studied monolithic zirconia crowns and three-unit FDPs in the literature.

A second limitation of the present study is the lack of a secondary higher force against Bruxzir. Considering that Bruxzir material was originally produced against bruxism, bruxzir would have exhibited more strength than 49 N. However, the applied force in the present study was 49 N which is accepted as a normal chewing force in the posterior region, was used for Bruxzir and Incoris TZI restorations. Further studies may be carried out considering chewing forces specific for bruxers.

## Conclusions

Based on the findings of this in vitro study, both the monolithic zirconia crowns showed a small but significantly increased volumetric loss compared to three-unit FDPs. Of the two tested monolithic systems, Incoris TZI exhibited greater wear than Bruxzir. The fracture strengths of Bruxzir crowns and FDPs were found to be greater than those of their counterparts fabricated with Incoris TZI. Bruxzir and Incoris TZI monolithic zirconia systems were found to be fracture-resistant for crowns and FDPs against physiologic chewing forces owing to dynamic aging in vitro test conditions.

## Abbreviations

%: Percent
3D: 3-Dimensional
FDP: Fixed dental prosthesis
g: Gram
h: Hour
min: Minute
ml: Milliliter
mm: Millimeter
mm^3^: Cubic millimeter
N: Newton
rpm: Revolutions per minute
wt: Weight
